# Self-Regulation and Regulatory Teaching as Determinants of Academic Behavioral Confidence and Procrastination in Undergraduate Students

**DOI:** 10.3389/fpsyg.2021.602904

**Published:** 2021-02-10

**Authors:** Jesús de la Fuente, Paul Sander, Angélica Garzón-Umerenkova, Manuel Mariano Vera-Martínez, Salvatore Fadda, Martha Leticia Gaetha

**Affiliations:** ^1^School of Education and Psychology, University of Navarra, Pamplona, Spain; ^2^School of Psychology, University of Almería, Almería, Spain; ^3^Department of Psychology, Teesside University, Middlesbrough, United Kingdom; ^4^School of Psychology, Fundación Universitaria Konrad Lorenz, Bogotá, Colombia; ^5^School of Psychology, University of Granada, Granada, Spain; ^6^Unit of Prevention of Stress, University of Sassari, Sassari, Italy; ^7^Universidad Popular Autónoma del Estado de Puebla, Puebla, Mexico

**Keywords:** theory of self-regulated learning vs. externally-regulated learning, academic behavioral confidence, procrastination, university, structural equation modeling

## Abstract

The combination of student Self-Regulation (SR) and the context of Regulatory Teaching (RT), each in varying degree, has recently been demonstrated to have effects on achievement emotions, factors and symptoms of stress, and coping strategies. The aim of the present research study is to verify its possible further effects, on academic behavioral confidence and procrastination. A total of 1193 university students completed validated online questionnaires with regard to specific subjects in their degree program. Using an ex post facto design, multivariate analyses and structural equation modeling (SEM) were carried out in order to test the relationships predicted by the model. SR and RT had a significant joint effect in determining the degree of academic behavioral confidence and of procrastination. Academic behavioral confidence also significantly predicted reasons for procrastinating, and these in turn predicted activities of procrastination. Conclusions are discussed, insisting on the combined weight of the two variables in determining academic behavioral confidence, reasons for procrastinating and activities subject to procrastination, in university students. Implications for guidance and educational support of university students and teachers are analyzed.

## Introduction

This study forms part of a series whose aim is to determine the combined effect of student self-regulation and of regulatory teaching on other academic variables. The aim of the present study, therefore, was to establish the combined effect of the student’s level of self-regulation (SR) and the level of regulatory teaching (RT) on students’ degree of academic behavioral confidence, as a precursor to reasons for procrastinating and to activities of procrastination. This study, then, would complete the body of published evidence that consistently indicates a joint effect of the two variables, self-regulation and regulatory teaching, in this Research Topic (de la Fuente et al., 2020a).

### The Teaching and Learning Process as Object of Study in Educational Psychology

In formal academic situations, such as the university, it seems reasonable that the variables we study would be jointly determined by learner characteristics as well as by the design and implementation of the teaching process (Cabanach et al., 2007, 2013; Chartier et al., 2011; Alonso-Tapia et al., 2018; Gentsch et al., 2018; Cassady et al., 2019). Previous theoretical models have adopted this idea. Biggs’ *3P model* (Biggs, 1989, 1993a, b, 1999a, b; Biggs et al., 2001) has evolved toward a more interactive vision, progressively integrating the teaching process more explicitly (Kember et al., 2020). The *Vermunt model* (Vermunt, 1995, 1996, 1998; Vermunt and Donche, 2017) has systematically analyzed the role of external regulation as a negative factor for appropriate learning styles (Vermunt, 2005, 2007). The *Entwistle model* (Entwistle and Ramsden, 1983; Entwistle, 1991) has specifically considered the weight of the context and teaching process in the university environment (Ramsden, 1991; Asikainen et al., 2014; Cano et al., 2020). *The Zimmerman* model (Zimmerman, 1990, 1998; Zimmerman and Schunk, 2001) has also considered the contextual factor, although in a more implicit way (Kim et al., 2020; Zalazar-Jaime and Medrano, 2020).

The Theory of Self- vs. Externally-Regulated Learning, *SRL vs. ERL Theory* (de la Fuente, 2017) has attempted to organize the different combinations of student regulation (learning process) and teacher regulation (teaching process) that can occur in a university academic setting, summarizing these in a five-combination heuristic (see Table 1). This heuristic assumes that each study variable should be contextualized within the teaching and learning process, representing a distinct approach to investigation in Educational Psychology. Assuming that the students and the teacher may have varying characteristics (high-medium-low in regulation), different combinations will result, and prove more or less favorable to the teaching and learning process:

**TABLE 1:** Combinations between model parameters hypothesized by SRL vs. ERL Theory (de la Fuente et al., 2017, 2019).

Combination Level		Regulation aver/rank	Regulation trend: Effect	Academic Behavioral Confidence*→	Procrastination*
SR Level (range)*	RT Level (range)*				
**3** (3.85–5.00) **H**	**3** (2.84–5.00) **H**	3.0/**5**	**High-High:** *High Regulation*	*High*	*Low*
**2** (3.10–3.84) **M**	**3** (2.84–5.00) **H**	2.5/**4**	**Medium-High**: *Regulation*	*M-H*	*M-L*
**3** (3.85–5.00) **H**	**2** (2.35–2.83) **M**	2.5/**4**	**High-Medium**: *Regulation*	*M-H*	*M-L*
**2** (3.10–3.84) **M**	**2** (2.35–2.83) **M**	2.0/**3**	**Medium:** *Non-Regulation*	*M*	*M*
**2** (3.10–3.84) **M**	**1** (1.00–2.34) **L**	1.5/**2**	**Medium-Low**: *Dysregulation*	*M-L*	*M-H*
**1** (1.00–3.09) **L**	**2** (2.35–2.83) **M**	1.5/**2**	**Low-Medium**: *Dysregulation*	*M-L*	*M-H*
**1** (1.00–3.09) **L**	**1** (1.00–2.34) **L**	1.0/**1**	**Low-Low:** *High Dysregulation*	*Low*	*High*

SR level and teaching level (L, low; M, medium; H, High); *effects analyzed in this investigation.

(1) The worst, *Very Unfavorable* combination (type 1) refers to a classroom combination of a student with low self-regulation (SR) and a teaching process low in external regulation (RT). In this case, the model predicts low academic behavioral confidence and high procrastination.
(2) An *Unfavorable* combination (type 2) refers to a classroom combination of a student with low SR and a teacher with medium RT, or the inverse. Here, the model predicts medium-low academic behavioral confidence and medium-high procrastination.
(3) A *Medium* combination (type 3) refers to the combination of a student with medium SR and a teacher with medium RT. The model predicts medium academic behavioral confidence and a medium level of procrastination.
(4) A *Favorable* combination (type 4) refers to the combination of a student with medium SR and a teacher with high RT, or the inverse. The model predicts medium-high academic behavioral confidence and a medium-low level of procrastination.
(5) The *Most Favorable* combination (type 5) refers to the combination of a student with high SR and a teacher with high RT. The model predicts high academic behavioral confidence and a low level of procrastination.

Previous research has consistently shown this heuristic to establish significant differences in the factors and symptoms of stress (de la Fuente et al., 2020a), coping strategies (de la Fuente et al., 2020b), achievement emotions (de la Fuente et al., 2019), students’ learning approaches (de la Fuente et al., 2020c) and academic achievement (de la Fuente et al., 2017). All these results are contextualized within the process of university teaching and learning (Gross, 2008, 2014, 2015a, b; Holinka, 2015; Harley et al., 2019; Hirvonena et al., 2019; Kobylińska and Kusev, 2019). Yet to be established, however, is its discriminatory power in determining the level of academic behavioral confidence and procrastination– two behavioral variables of learning that are polar opposites in their association with self-regulated learning at university. Consequently, this will be the aim of the present study.

### Academic Behavioral Confidence as Variable of the Teaching and Learning Process

#### Academic Behavioral Confidence as a Variable of the Learning Process

Although *academic behavioral confidence* has been defined as an eminently personal and attitudinal construct (Sander and Sanders, 2009; Sander et al., 2013), its self-referring, subjective, perceptual nature suggests that it can be influenced by both personal and contextual factors. Previous research has reported that *academic behavioral confidence* is associated with and is a positive predictor of a deep learning approach and of academic achievement (de la Fuente et al., 2016). Moreover, it has been positively associated with self-regulation (Nicholson et al., 2013; de la Fuente et al., 2015b), and has a stable nature, associated with academic goals (Putwain et al., 2013). Another research report has shown the predictive value of academic confidence on academic performance (Burr and LeFevre, 2020). Academic confidence has also appeared as a predictor of coping strategies and achievement (Kirikkanat and Kali-Soyer, 2018), as well as predicting confidence in learning (Shoemaker, 2010). More recently, it has been found in association with and a positive predictor of positive achievement emotions, as well as negatively predicting negative emotions (Sander and de la Fuente, 2020).

Earlier research in the development of the academic behavioral confidence scale has shown that the scale meaningfully discriminates between students in different degree programs, such that students in programs that require higher grades at entry, for example Medicine, Speech and Language Therapy, and Nutrition have higher confidence in one or more of the Grades, Studying and Attendance sub-scales (Sander and Sanders, 2009). In a summary article, Sander (2009) presents findings that indicate that dyslexic students studying at universities in the United Kingdom have lower academic confidence on the Grades, Verbalizing and Studying sub-scales but not on the Attendance sub-scale. Furthermore, other data from United Kingdom university students shows that scores on the academic behavioral confidence scale drop during a course of study (Sander, 2009; Putwain and Sander, 2016), a finding that is supported by other research (Beyer, 1999; Zusho et al., 2003; Papinczak et al., 2008).

#### Academic Behavioral Confidence as a Variable Promoted Through Teaching

Prior research has demonstrated that level of regulatory teaching determined the degree of academic behavioral confidence (de la Fuente et al., 2015b). However, we have not yet seen whether academic behavioral confidence is determined linearly and jointly both by student characteristics and teaching process characteristics (Akbari and Sahibzada, 2020). A pertinent factor to be considered is that the grades and verbalizing components of academic behavioral confidence are under the control of the student only to a lesser degree, whereas the studying and attendance components are largely under the student’s control (Sander, 2009; Sander and Sanders, 2009). A student may choose to study or attend whereas the grades one receives depends partly on the marker, and one’s experience of discussing course materials depends on the person one is talking to. As Putwain and Sander (2016) say, “The dip and return of confidence in studying and attendance may reflect a closer alignment with self-regulative processes determined by control than grades and verbalizing” (p. 393). Finally, show how the expectations that students have of their and their teachers’ responsibility in the teaching and learning process interact with student academic confidence in the prediction of grades.

### Procrastination as a Variable of the Teaching and Learning Process

#### Procrastination as a Variable of the Learning Process

Procrastination has been studied and described for general matters of daily life as well as for specific areas, such as the contexts of health and academics. Procrastination is understood to be a failure in motivation that creates a gap between intention and action, with negative consequences for the individual (Steel, 2007; Steel and Klingsieck, 2015), and has been established as the polar opposite of self-regulation. It has thus been considered a dysregulatory behavior (de la Fuente, 2017), being negatively predicted by self-regulation (Garzón-Umerenkova et al., 2018). High levels of procrastination have also been related to anxiety problems, general stress, and physical and mental health issues (Stead et al., 2010; Sirois and Tosti, 2012; Kim and Seo, 2015). In general, research studies on procrastination can be classified as focusing either on the reasons that lead to procrastinating, or on the activities or frequency of procrastination behaviors. The *motives* that define the volitional basis leading to procrastination differ in valence (positive vs. negative emotionality) and direction (approach vs. avoidance), while frequency describes the intensity of procrastination in different activities.

The study of the *motives* or *reasons for procrastinating* has established certain commonalities, such as attraction/uncertainty about the task, fear of failure or fear of evaluation (Zarick and Stonebraker, 2009) and perfectionism (Sudler, 2013). Among university students, inadequate time management, test anxiety, and laziness are the principal triggers for procrastinating (Gil et al., 2019).

Examples of procrastination in activities of *daily life* may involve paying a bill or taking one’s medication; in the academic context, preparing for a test or doing an assignment. General procrastination behaviors have been on the rise in recent decades. In the 1970s, figures for recurring procrastinators fell between 4–5% of the adult population, while this incidence has recently been estimated at 15–20% (Steel and Ferrari, 2012). Specifically, academic procrastination appears with greater frequency than general procrastination. Certain studies indicate that students often put off starting to prepare for exams (30–40%) or writing papers (46%) (Rothblum et al., 1986; Beswick et al., 1988).

At the same time, the *intensity* of academic procrastination shows differences between certain population subgroups. For example, gender has been described as having an indirect effect on procrastination and academic performance, with lower levels of procrastination and greater achievement in women; age also has an effect, where procrastination is positively predicted in younger people (Garzón-Umerenkova et al., 2018). Similarly, there is evidence to indicate that procrastination varies according to the student’s degree program (Clariana, 2013); that there is a greater tendency to procrastinate in the transition from high school to university; and that procrastination is associated with plagiarism or dishonest academic behavior (Clariana et al., 2012).

Different studies have confirmed that procrastination is inversely associated with academic achievement: the greater the procrastination, the lower the achievement (Kim and Seo, 2015). Procrastination has more predictive value for achievement than do variables like class attendance or university admissions scores (Steel, 2007; Rozental and Carlbring, 2014). Procrastination has also been associated with other important academic variables. When students perceive tasks as difficult, unattractive, ambiguous and requiring more effort, they tend to present higher rates of procrastination (Ferrari et al., 2006). Accordingly, those who present more confidence in their academic skills (high levels of self-efficacy) tend to procrastinate less, and procrastination has less impact on their academic achievement (Klassen et al., 2008).

The psychological mechanism by which procrastination originates in the student seems to be low *expectation of achievement;* this affects motivation to start the task and to follow through, possibly leading to avoidance behavior and procrastination (Rozental and Carlbring, 2014). *Self-efficacy* seems to mediate the effect of achievement expectations; students with low perceived self-efficacy are more vulnerable to being caught in a vicious cycle of procrastination (Wäschle et al., 2014). By contrast, high levels of self-efficacy are related to the use of planning tools and starting tasks at the right time (Wolters, 2003).

#### Procrastination as a Variable Promoted Through the Teaching Process

Given that the mechanism behind this behavioral phenomenon is a lack of motivation or expectations, it is reasonable that most research has focused on procrastination as it relates to student characteristics, looking for internal explanatory mechanisms. However, it is also possible that procrastination can be triggered externally, by characteristics of the teaching process (Codina et al., 2020; Yang, 2020). Insufficient attention has been given to this perspective. Adopting the perspective of SRL vs. ERL Theory (de la Fuente, 2017) allows us to take this two-fold approach.

Situational and contextual factors –social factors included– play an important role in explaining the types of procrastination. Parents’ and teachers’ negative attitudes toward procrastination, for example, have been found to trigger a kind of procrastination as rebellion (Klingsieck et al., 2013). There is evidence that students’ perception of autonomy-supportive teaching, or effective or regulatory teaching, is positively associated with feeling competent, and negatively associated with procrastination behaviors (Codina et al., 2020). Procrastination increases when the teacher lowers demands, is willing to negotiate academic deadlines, and tends to be more flexible in grading (Schraw et al., 2007). Consequently, task characteristics and teacher characteristics, as powerful contextual factors, are important in triggering or increasing the likelihood of procrastination in students (Steel and Klingsieck, 2016).

### Aims and Hypotheses

Based on the models and previous empirical data, the following research objectives were set: (1) to establish whether the combination levels defined in SRL vs. ERL Theory (Table 1) determine the level of academic behavioral confidence, as well as reasons for and activities of procrastination; (2) to determine the predictive value of both self-regulation and regulatory teaching in academic behavioral confidence, and the latter’s predictive value in reasons for and activities of procrastination.

From these objectives, the following *hypotheses* were stated. (1) A *graded increase in level of regulation* (internal and external) would give rise to an increase in academic behavioral confidence, and a proportionate decrease in reasons for and activities of procrastination. By contrast, a *graded decrease in level of regulation* (internal and external) would give rise to a decrease in academic behavioral confidence, and a proportionate increase in reasons for and activities of procrastination. (2) Regulation factors in students and in the teaching would be positive, significant predictors of academic behavioral confidence; the latter would in turn negatively predict reasons for and activities of procrastination.

## Materials and Methods

### Participants

To establish interdependence relations between low-medium-high levels of *Self-Regulation* (SR) and *Regulatory Teaching* (RT), we used a total sample of 1193 undergraduate students from two public universities of Spain, taken through convenience sampling. The sample contained students majoring in Psychology, Primary Education, and Educational Psychology; 85.5% were women and 14.5% were men. The age range was 19 to 25 years, and mean age was 21.33 (σ = 2.26) years.

### Instruments

#### Self-Regulation (Meta-Behavioral Variable)

The *Short Self-Regulation Questionnaire* (SSRQ) (Miller and Brown, 1991) was used to measure this variable. The Spanish version has been validated in Spanish samples (Pichardo et al., 2014, 2018), showing acceptable validity and reliability values, comparable to the English version. The Spanish Short SRQ comprises four factors (goal setting-planning, perseverance, decision making and learning from mistakes) and contains 17 items (all with saturations greater than 0.40). This questionnaire has a Likert format, with possible responses ranging from 1 (“not true of me at all”) to 5 (“very true of me). It has the advantage of significantly reducing completion time with respect to the original 63-item scale. The confirmatory factor structure is consistent (Chi-Square = 250.83, df = 112, CFI = 0.90, GFI = 0.92, AGFI = 0.90, RMSEA = 0.05). Internal consistency was acceptable for the questionnaire total (α = 0.86) and for all factors: goal setting-planning (α = 0.79), decision making (α = 0.72), learning from mistakes (α = 0.72), and perseverance (α = 0.73). Correlations were obtained for the following: (1) between each item and its factor total, (2) between the factors, and (3) between each factor and the questionnaire total. The results were good in all cases, except for decision making, which had a lower correlation with other factors (0.41 to 0.58). The correlations between the original long SRQ and the long Spanish version, and between the English short SRQ and the Spanish short version are better for the short version (original SSRQ: *r* = 0.85 and Spanish SSRQ: *r* = 0.94; *p* < 0.01) than for the original, long SRQ (*r* = 0.79; *p* < 0.01).

#### Regulatory Teaching (Meta-Instructional Variable)

The *Assessment of the Teaching-Learning Process, ATLP, student version* (de la Fuente et al., 2012) was used to evaluate students’ perception of the teaching process. The Regulatory Teaching scale constitutes Dimension 1 of the confirmatory model. The ATLP-D1 contains 29 items with a five-factor structure: Specific regulatory teaching, regulatory assessment, preparation for learning, satisfaction with the teaching, and general regulatory teaching. Having been previously validated in university students (de la Fuente et al., 2012, 2020c), the scale shows a factor structure with adequate fit indices (Chi-Square = 590.626; df = 48, *p* < 0.001, CFI = 0.938, TLI = 0.939, NFI = 0.950, NNFI = 0.967; RMSEA = 0.058). Internal consistency is also adequate (ATLP D1: α = 0.83; specific regulatory teaching, α = 0.897; regulatory assessment, α = 0.883; preparation for learning, α = 0.849; satisfaction with the teaching, α = 0.883 and general regulatory teaching, α = 0.883). The ATLP is a self-report instrument that collects data from students and teachers and is available in Spanish and English. External validity results are also consistent, since there are several interdependent relationships between the reported perceptions of variables in an academic setting.

#### Academic Behavioral Confidence (Attitudinal Variable)

This was measured by the Academic Behavioral Confidence Scale (Sanders and Sander, 2003; Sander and Sanders, 2006, 2009) in a validated Spanish version (Sander et al., 2011). Developed from the established constructs of self-concept and self-efficacy, the ABC scale assesses specific aspects in undergraduate students. This psychometric scale, designed for students from Spain and the United Kingdom, asks them to report their anticipated study-related behaviors within their degree program (assumed to consist primarily of lecture-based courses). Crucially distinct aspects of students’ academic behavior are represented in four subscales: Grades, Studying, Verbalizing and Attendance (Sander, 2009). Students are required to respond to a question stem (‘How confident are you that you will be able to…’) for items such as ‘…manage your workload to meet coursework deadlines’ and ‘…write in an appropriate academic style.’ Responses fall along a five-point scale (1 = ‘not at all confident,’ 5 = ‘very confident’). A higher score therefore indicates greater confidence in one’s efficacy in study skills or behaviors. A four-factor model (confidence in attaining grades, studying, attending classes and discussing course material) has shown adequate reliability and validity in prior studies (Sander and Sanders, 2009). The confirmatory model showed good fit [Chi-square = 693.405; Degrees of freedom (152–54) = 98; *p* ≤ 0.001; NFI = 0.916; RFI = 0.904; IFI = 0.927; TLI = 0.909, CFI = 0.927; RMSEA = 0.062; HOELTER = 276 (*p* < 0.05) and 302 (*p* < 0.01)]. There is also good internal consistency for the total scale [α = 0.952; Part 1 = 0.932, Part 2 = 0.872; Spearman-Brown = 0.961; Guttman = 0.935].

#### Procrastination (Motivational Variable)

Procrastination Assessment Scale-Students, in its Spanish version (Garzón and Gil, 2017). This scale was originally constructed by Solomon and Rothblum (1984) and has been often used in the study of academic procrastination internationally. Its 44 items describe the frequency of academic procrastination activities (18 items) and the underlying reasons for doing them (26 items). Thirteen possible reasons for procrastinating are incorporated, including such options as: evaluation anxiety, perfectionism, difficulty making decisions, dependency and help seeking, aversiveness of the task, lack of self-confidence and laziness. Response options are presented on a Likert scale with values from 1 to 5, where 1 means “does not reflect my motives at all,” 3 means “it reflects them to a certain degree” and 5 means “it reflects them perfectly.”

For the present study, we considered procrastination frequency in the academic activities addressed by the PASS: writing a term paper, studying for an exam, keeping up with weekly assigned reading, performing administrative tasks, attendance. Each activity also included the question: To what degree is procrastination in this area a problem for you? and, To what degree would you like to decrease your procrastination in this area? For this section, the test uses a five-point Likert scale: 1 (Never), 2 (Almost never), 3 (Sometimes), 4 (Almost always) and 5 (Always). Reasons for procrastinating were grouped into five factors: arousal seeking, low self-control, perfectionism, test anxiety and low self-confidence.

### Procedure

Participants voluntarily completed the scales using an online platform (de la Fuente et al., 2015a). The assessments covered a total of five specific teaching-learning processes of different university subjects over a period of two academic years. All the questionnaires were answered in their Spanish versions, previously translated and validated, using the online platform^1^. This research platform allows teachers and students to register online and give their informed consent. Each questionnaire is completed independently; students then have access to their scores for the total construct and for its factors. Additionally, the student can access self-help feedback, based on their scores, to work on aspects of their learning process. This platform is presently available in Spanish and English, but the number of available languages for questionnaire completion is currently being expanded, following validation of each tool in each language. *Self-regulation* and *Academic Behavioral Confidence* were evaluated in October-November of 2018 and 2019; *Procrastination Behavior and Regulatory Teaching* in March-April 2018 and 2019.

Students signed their informed consent and received a certificate of Project participation for completing the inventories outside of regular class hours. The procedure was approved by the respective Ethics Committees of the two universities, in the context of an R&D Project (see Funding).

### Data Analysis

#### Research Design

In line with the method of sample selection, an *ex post facto* design was used, collecting the data and manipulating it by selection.

##### Inferential effects of regulation levels

Through cluster analysis, continuous independent variables were transformed into discrete dependent variables, with three levels (low-medium-high). Preliminary analyses were carried out to determine the distribution of the variables, and so be able to perform analyses of variance [SR (*M* = 3.48, *SD* = 0.60); Kolmogoroff-Smirnoff = 0.25, *p* < 0.200; RT (*M* = 3.37, *SD* = 0.59); Kolmogoroff-Smirnoff = 0.37, *p* < 0.3501]. ANOVAs and MANOVAs were conducted, with Self-Regulation and Regulatory Teaching as independent Variables (IV), while Academic Behavioral Confidence and Procrastination were the dependent Variables. In all cases, error variance differences were confirmed to be non-significant (Box’s M test as a multivariate statistical test used to check the equality of multiple variance-covariance matrices. The test is commonly used to test the assumption of homogeneity of variances and covariances in MANOVA and linear discriminant analysis), *p* > 0.05). The multivariate analyses (MANOVAs) showed a statistically significant main effect of the five interaction types on low-medium-high levels of the dependent variables (see Table 1):

*Combination 1* represents a statistically significant low level of SR and low level of RT (1 and 1). The average regulation level is 1.0, and its regulation rank is 1. The regulation trend is low SR and low RT; this is associated with a high level of dysregulation. The effects would be a low level of academic behavioral confidence and a high level of procrastination reasons and activities.

*Combination 2* represents a statistically significant low level in SR and medium level in RT, or vice versa (1 and 2, or 2 and 1). The average regulation level is 1.5, and its regulation rank is 2. The regulation trend is low SR and medium RT, or vice versa; associated in turn with a medium-low level of dysregulation. The effects, then, would be a low-medium level of academic behavioral confidence and a medium-high level of procrastination reasons and activities.

*Combination 3* represents a statistically significant medium level of SR and medium level of RT (2 and 2). The average regulation level is 2.0, and its regulation rank is 3. The regulation trend is medium SR and medium RT; this is associated with a medium level of dysregulation. The effects, then, would be a medium level of academic behavioral confidence and a medium level of procrastination reasons and activities.

*Combination 4* represents a statistically significant medium level in SR and high level in RT, or vice versa (2 and 3, or 3 and 2). The average regulation level is 2.5, and its regulation rank is 4. The regulation trend is high SR and medium RT, or medium SR and high RT; this is associated with a good level of regulation. The effects, then, would be a medium-high level of academic behavioral confidence and a medium-low level of procrastination reasons and activities.

*Combination 5* represents statistically significant high levels of SR and RT (3 and 3). The average regulation level is 3.0, and its regulation rank is 5. The regulation trend is high SR and high RT; this is associated with a high level of regulation. The effects, then, would be a high level of academic behavioral confidence and a low level of procrastination reasons and activities.

##### Predictive structural effects

For analysis of SEM model fit, the comparative adjustment index (CFI) and the mean square approximation error (RMSEA) were used. CFI values equal to or greater than 0.90 and 0.95, respectively, were taken to indicate acceptable and close fit to the data (McDonald and Marsh, 1990). RMSEA values equal to or less than 0.08 and 0.05 were also taken to indicate acceptable and close levels of fit (Jöreskog and Sörbom, 1993). IBM-AMOS statistical program (v. 22) was used.

## Results

### Interdependent Complex Effects Between Levels of Self-Regulation (SR) and Levels of Regulatory Teaching (RT)

#### Effect on Total Academic Behavioral Confidence and Its Factors

There was a statistically significant main effect of SR levels (1 = low; 2 = medium; 3 = high) on total *Academic Behavioral Confidence* (1 < 2 < 3, *p* < 0.001). Complementarily, there was a statistically significant main effect of RT (1 = low; 2 = medium; 3 = high) on total *Academic Behavioral Confidence* (1 < 2 < 3, *p* < 0.001). A statistically significant effect of SR levels and RT levels was noted in all factors of *Academic Behavioral Confidence*. There was no statistically significant SR × RT interaction effect. The most powerful effect of SR was produced on the factors of *Grades* and *Verbalization*, while the most powerful effect of RT was on the factors of *Grades, Verbalization*, and *Attendance.* See Table 2.

**TABLE 2:** Interdependent complex effects (3 × 3) of low-medium-high levels of *Self-Regulation (SR)* and low-medium-high levels of *Regulatory Teaching (RT)* with academic behavioral confidence and procrastination (*n* = 986).

SR	Low(*n* = 246)			Medium(*n* = 473)			High(*n* = 267)			Variable	F(Pillai’s)	*post-hoc* effects
RT	Low	Med	High	Low	Med	High	Low	Med	High			
*N*=	58	134	54	85	230	158	29	102	136			
**Academic Behavioral Confidence**												
Total	3.13 (0.64)	3.40 (0.50)	3.49 (0.51)	3.54 (0.48)	3.66 (0.44)	3.86 (0.50)	3.89 (0.51)	4.00 (0.44)	4.22 (0.44)	SR	*F*(2,957) = 98.987**, *n*^2^ = 0.171;	pow = 1,0; 1 < 2 <3**
										RT	*F*(2,957) = 19.795**, *n*^2^ = 0.040;	pow = 1,0; 1 < 2 < 3**
Factors										SR	*F*(8,1981) = 31.307**; *n*^2^ = 0.116;	
										RT	*F*(8,1981) = 7.301**; *n*^2^ = 0.030	
F1. Grades	3.41 (0.76)	3.75 (0.50)	3.87 (0.53)	4.03 (0.50)	4.01 (0.45)	4.16 (0.18)	4.20 (0.43)	4.33 (0.43)	4.52 (0.41)	SR*	*F*(2,957) = 87.830**, *n*^2^ = 0.115;	pow = 1.0; 1 < 2 < 3**
										RT*	*F*(2,957) = 18.192**, *n*^2^ = 0.037,	pow = 1.0; 1 < 2 < 3**
F2. Verbalization	3.16 (0.75)	3.47 (0.59)	3.63 (0.56)	3.64 (0.54)	3.85 (0.51)	4.01 (0.57)	4.14 (0.63)	4.21 (0.47)	4.47 (0.53)	SR*	*F*(2,957) = 119.302**, *n*^2^ = 0.200;	pow = 1.0; 1 < 2 < 3**
										RT*	*F*(2,957) = 18.985**, *n*^2^ = 0.038;	pow = 1.0; 1 < 2 < 3**
F3. Study	2.62 (0.89)	2.78 (0.97)	2.85 (0.97)	2.97 (0.97)	3.01 (0.98)	3.28 (0.89)	4.14 (0.63)	4.21 (0.47)	4.44 (0.43)	SR	*F*(2,957) = 31.389**, *n*^2^ = 0.062;	pow = 1.0; 1 < 2 < 3**
										RT	*F*(2,957) = 3.525*, *n*^2^ = 0.007;	pow = 0.625; 1,2 < 3**
F4. Attendance	3.24 (0.95)	3.62 (0.71)	3.70 (0.71)	3.72 (0.76)	3.86 (0.75)	4.00 (0.61)	3.87 (0.70)	4.03 (0.62)	4.22 (0.64)	SR	*F*(2,957) = 28.606**, *n*^2^ = 0.056;	pow = 1.0; 1 < 2 < 3**
										RT*	*F*(2,957) = 13.737**, *n*^2^ = 0.027;	pow = .998; 1,2 < 3**
**Procrastination**												
**Reasons for Procrastination**												
Total	3.00 (0.49)	2.63 (0.56)	2.57 (0.91)	2.41 (0.59)	2.29 (0.58)	2.20 (0.61)	2.25 (0.57)	2.11 (0.60)	1.83 (0.47)	SR*	*F*(2,202) = 13.022**, *n*^2^ = 0.114;	pow = 0.997; 1 > 2 > 3**
										RT	*F*(2,202) = 3.083*, *n*^2^ = 0.030;	pow = 0.590; 1 > 2,3**
Factors												
R1. Arousal seekg	3.10 (0.77)	2.97 (0.75)	2.82 (1.0)	2.50 (0.85)	2.48 (0.68)	2.40 (0.85)	2.20 (0.75)	2.12 (0.67)	2.06 (0.78)	SR	*F*(2, 202) = 10.837**, *n*^2^ = 0.097;	pow = 0.990; 1,2 > 3**
										RT	*F*(2, 202) = 0.182^ns^, *n*^2^ = 0.002,	pow = 0.078;
R2. L. Self-control	3.56 (0.90)	3.35 (0.92)	2.85 (1.0)	2.88 (1.0)	2.72 (.79)	2.28 (0.94)	2.65 (0.94)	2.23 (0.93)	2.03 (0.92)	SR*	*F*(2, 202) = 10.992**, *n*^2^ = 0.098;	pow = 0.990; 1 > 2 > 3**
										RT*	*F*(2, 202) = 5.337**, *n*^2^ = 0.050;	pow = 0.836; 1 < 2,3**
R3. Perfectionism	3.34 (0.51)	2.89 (0.76)	2.66 (1.0)	2.76 (0.82)	2.59 (0.72)	2.51 (0.51)	2.56 (0.71)	2.34 (0.70)	2.32 (0.67)	SR*	*F*(2,202) = 6.111**, *n*^2^ = 0.057;	pow = 0.684; 1 > 2 > 3*
										RT	*F*(2,202) = 2.201^ns^, *n^2^* = 0.021;	pow = 0.446
R4. Test anxiety	2.77 (0.78)	2.28 (1.1)	2.33 (1.3)	1.94 (1.0)	2.18 (1.0)	1.88 (1.0)	2.60 (0.96)	1.97 (0.78)	1.32 (0.60)	SR*	*F*(2,202) = 3.943* *n*^2^ = 0.038;	pow = 0.704; 1 > 2,3**
										RT*	*F(*2,202) = 4.009*, *n*^2^ = 0.038;	pow = 0.712; 1 > 3*
R5. Low Confidence	2.12 (0.89)	1.85 (0.83)	2.08 (0.92)	1.63 (0.79)	1.82 (0.76)	1.79 (0.72)	1.33 (0.77)	1.88 (0.89)	1.42 (0.69)	SR	*F*(2,202) = 3.375*, *n*^2^ = 0.032;	pow = 0.632; 1 > 3*
										RT	*F*(2,202) = 1.574^ns^, *n*^2^ = 0.030;	pow = 0.481; 1 > 3*
**Procrastination Activities**												
Total	3.00 (0.49)	2.63 (0.56)	2.50 (0.91)	2.31 (0.59)	2.27 (0.57)	2.20 (0.61)	2.25 (0.57)	2.11 (0.60)	1.83 (0.47)	SR*	*F*(2,202) = 13.022**, *n*^2^ = 0.114;	pow = 0.997; 1 > 2 > 3**
										RT	*F*(2,202) = 3.083*, *n*^2^ = 0.030;	pow = 0.590; 1,2 > 3**
Factors										SR*	*F*(12,442) = 2.507**, *n*^2^ = 0.067;	pow = 0.828;
										RT	*F*(12,442) = 1.569*, *n*^2^ = 0.050;	pow = 0.590;
F1. Term papers	3.71 (0.78)	3.84 (0.70)	3.66 (0.94)	3.70 (0.55)	3.36 (0.73)	3.19 (0.81)	2.33 (1.0)	3.20 (0.83)	3.13 (0.73)	SR*	*F*(2,215) = 12.550**, *n*^2^ = 0.105;	pow = 0.996; 1 > 2 > 3**
										RT	*F*(2,215) = 1.220^ns;^ *n*^2^ = 0.001;	pow = 0.246; 1 > 3*
F2. Study for exams	3.87 (0.75)	4.10 (0.56)	3.97 (0.76)	3.83 (0.74)	3.52 (0.80)	3.28 (1.0)	2.93 (1.4)	3.31 (0.99)	3.11 (1.0)	SR*	*F*(2,215) = 8.582**; *n*^2^ = 0.074;	pow = 0.966; 1 > 2 > 3**
										RT	*F*(2,215) = 0.873^ns;^ *n*^2^ = 0.008;	pow = 0.211; 1,2 > 3*
F3. Assigned rdg.	3.77 (0.78)	3.64 (0.76)	3.55 (1.0)	3.55 (1.0)	3.67 (0.86)	3.26 (1.0)	2.66 (1.3)	3.23 (1.0)	2.94 (0.95)	SR*	*F*(2,215) = 6.619**; *n*^2^ = 0.058;	pow = 0.909; 1 > 2,3**
										RT	*F*(2,215) = 1.585^ns^; *n*^2^ = 0.015;	pow = 0.334; 1,2 > 3*
F4. Admin. tasks	2.80 (0.87)	3.14 (1.0)	2.91 (1.0)	2.44 (0.99)	2.81 (1.0)	2.40 (1.1)	2.60 (1.36)	2.68 (1.1)	2.24 (1.1)	SR	*F*(2,215) = 2.579*; *n*^2^ = 0.023;	pow = 0.511; 1 > 3**
										RT	*F*(2,215) = 2.163^ns^; *n*^2^ = 0.020;	pow = 0.440; 1,2 > 3*
F5. Attendance	3.35 (0.87)	3.18 (1.0)	3.33 (1.2)	3.08 (0.73)	3.07 (0.98)	2.66 (1.5)	2.80 (1.1)	2.88 (1.0)	2.75 (1.1)	SR	*F*(2,215) = 2.746*; *n*^2^ = 0.025;	pow = 0.538; 1 > 3**
										RT	*F*(2,215) = 0.382^ns^; *n*^2^ = 0.004;	pow = 0.111;
F6. Active in general	3.33 (0.72)	2.97 (0.93)	3.50 (1.0)	3.18 (0.73)	3.00 (0.99)	2.69 (0.96)	3.20 (1.3)	2.72 (1.0)	2.61 (0.96)	SR	*F*(2,215) = 2.477*; *n*^2^ = 0.023;	pow = 0.494; 1 > 3**
										RT	*F*(2,215) = 1.487^ns^; *n*^2^ = 0.014;	pow = 0.315;

*Statistical effect with higher *F* value: featured effect. *p < 0.05; **p < 0.01; ***p < 0.001.

#### Effect on Total Reasons for Procrastination and Its Factors

**FIGURE 1:**
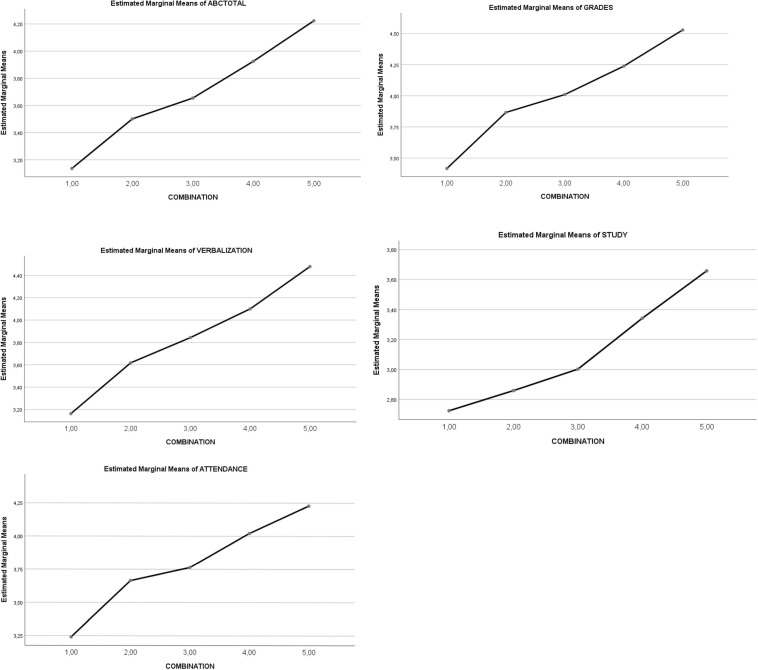
Graphical representation of the effect of combination types (1–5) on *Academic Behavioral Confidence*.

A statistically significant main effect of SR levels (1 = low; 2 = medium; 3 = high) was noted on total *Reasons for Procrastination* (1 > 2 > 3, *p* < 0.001). Complementarily, a statistically significant main effect of RT levels (1 = low; 2 = medium; 3 = high) was noted on total *Reasons for Procrastination* (1,2 > 3, *p* < 0.001). Complementary, a statistically significant main effect of SR levels was noted on the factors of *Reasons for Procrastination.* The main partial effects of SR appeared in the procrastination reasons of *low self-control*, *perfectionism* and *test anxiety* (1 > 2 > 3, *p* < 0.001), while the main partial effects of RT appeared in the reasons *low self-control* and *test anxiety* (1 > 2 > 3, *p* < 0.001). See Table 3 and Figure 1.

**TABLE 3:** Effects of combination types on academic behavioral confidence, procrastination reasons and activities (*n* = 1026).

	Combination Types (IVs)					
	1	2	3	4	5	*post-hoc* effects
	(*n* = 63)	(*n* = 236)	(*n* = 338)	(*n* = 253)	(*n* = 140)	
**DVs**						
Configuration Group						*F*(4,1025) = 421.752*** (Pillai, *n*^2^ = 0.622; pow = 1.0
*GRUP-Self-Regulation*	1.00 (0.00)	1.38 (0.48)	1.92 (0.51)	2.43 (0.49)	3.00 (0.00)	*F*(4,1025) = 421.752***, *n*^2^ = 0.622, pow = 1.0; all; *p* < 0.001
*GRUP-Regulatory Teaching*	1.00 (0.00)	1.61 (0.48)	2.07 (0.51)	2.56 (0.49)	3.00 (0.00)	*F*(4,1025) = 370.801**, *n*^2^ = 0.591, pow = 1.0; all *p* < 0.001
**Academic Behavioral Confidence**						
Total	3.13 (0.64)	3.50 (0.50)	3.65 (0.47)	3.92 (0.48)	4.22 (0.44)	*F*(4,961) = 78.261**; *n*^2^ = 0.246; pow = 1.0; 5,4 > 3,2 > 1**
Factors						*F*(16,3844) = 20.745**; *n*^2^ = 0.079; pow = 1.0
F1. Grades	3.41 (0.76)	3.86 (0.52)	4.01 (0.47)	4.23 (0.50)	4.52 (0.41)	*F*(4,961) = 67.994**; *n*^2^ = 0.221; pow = 1.0; 5,4 > 3,2 > 1**
F2. Verbalization	3.16 (0.75)	3.61 (0.59)	3.84 (0.54)	4.10 (0.53)	4.77 (0.43)	*F*(4,961) = 84.236**; *n*^2^ = 0.260; pow = 1.0; 5 > 4 > 3,2 > 1**
F3. Study	2.72 (0.89)	2.85 (0.97)	3.00 (0.85)	3.34 (0.87)	3.65 (0.89)	*F*(4,961) = 24.558*; *n*^2^ = 0.093; pow = 1.0; 5,4 > 3,2,1**
F4. Attendance	3.24 (0.95)	3.66 (0.73)	3.76 (0.66)	4.02 (0.61)	4.22 (0.65)	*F*(4,961) = 30.354**; *n*^2^ = 0.112; pow = 1.0; 5,4 > 3 > 2,1**
**Reasons for Procrastination**						
Total	3.00 (0.49)	2.47 (0.57)	2.42 (0.65)	2.16 (0.60)	1.83 (0.65)	*F*(4,206) = 11.080**; *n*^2^ = 0.177; pow = 1.0; 1 > 2,3 > 4,5**
Factors						*F*(20,802) = 3.381**; *n*^2^ = 0.076; pow = 1.0;
R1. Arousal	3.10 (0.77)	2.64 (0.81)	2.54 (0.81)	2.32 (0.79)	2.06 (0.78)	*F*(4,206) = 5.056**; *n*^2^ = 0.089; pow = 0.962; 1 > 2,3 > 4,5**
R2. Low Self-control	3.56 (0.90)	3.11 (1.0)	2.74 (0.84)	2.26 (0.93)	2.03 (0.92)	*F*(4,206) = 12.184**; *n*^2^ = 0.191; pow = 1.0; 1 > 2,3 > 4,5**
R3. Perfectionism	3.43 (0.51)	2.74 (0.80)	2.70 (0.78)	2.43 (0.76)	2.32 (0.67)	*F*(4,206) = 5.577**; *n*^2^ = 0.101; pow = 0.981; 1 > 2,3 > 4,5**
R4. Test anxiety	2.77 (0.78)	2.11 (1.0)	2.25 (1.0)	1.91 (0.83)	1.32 (0.60)	*F*(4,206) = 7.241**; *n*^2^ = 0.123; pow = 1.0; 1 > 2,3 > 4,5**
R5. Low Confidence	2.12 (0.89)	1.83 (0.81)	1.74 (0.79)	1.70 (0.69)	1.42 (0.69)	*F*(4,206) = 2.227*; *n*^2^ = 0.042; pow = 0.658; 1 > 2,3,4 > 5*
**Activities of Procrastination**						
Total	3.45 (0.61)	3.41 (0.51)	3.20 (0.71)	2.91 (0.72)	2.80 (0.70)	*F*(4,219) = 7.257**; *n*^2^ = 0.177; pow = 0.997; 1,2 > 3 > 4,5**
Factors						*F*(24,868) = 1.815**; *n*^2^ = 0.048; pow = 0.880
F1. Term papers	3.71 (0.78)	3.67 (0.63)	3.33 (0.85)	3.19 (0.81)	3.13 (0.73)	*F*(4,219) = 6.174**; *n*^2^ = 0.101; pow = 0.978; 1 > 2,3 > 4,5*
F2. Study for exams	3.77 (0.75)	3.67 (0.67)	3.56 (0.33)	3.30 (1.0)	3.11 (1.0)	*F*(4,219) = 6.604**; *n*^2^ = 0.108; pow = 0.991; 1,2 > 3,4 > 5**
F3. Assigned reading	3.77 (0.76)	3.71 (0.76)	3.27 (1.0)	3.07 (0.96)	2.94 (0.95)	*F*(4,219) = 5.974**; *n*^2^ = 0.098; pow = 0.984; 1,2 > 3 > 4,5**
F4. Admin. tasks	2.80 (0.87)	2.78 (1.0)	2.81 (1.0)	2.52 (1.1)	2.24 (1.1)	*F*(4,219) = 2.163*; *n*^2^ = 0.038; pow = 0.633; 1,2,3 > 4 > 5*
F5. Attendance	3.35 (0.87)	3.31 (0.91)	3.10 (1.0)	2.76 (1.0)	2.73 (1.1)	*F*(4,219) = 2.110*; *n*^2^ = 0.037; pow = 0.621;
F6. Activities in general	3.33 (0.72)	3.07 (0.84)	3.05 (0.07)	2.70 (1.1)	2.61 (0.96)	*F*(4,219) = 3.363**; *n*^2^ = 0.058; pow = 0.842;

*p < 0.05; **p < 0.01; ***p < 0.001.

#### Effect on Total Procrastination Behavior and Its Factors

**FIGURE 2:**
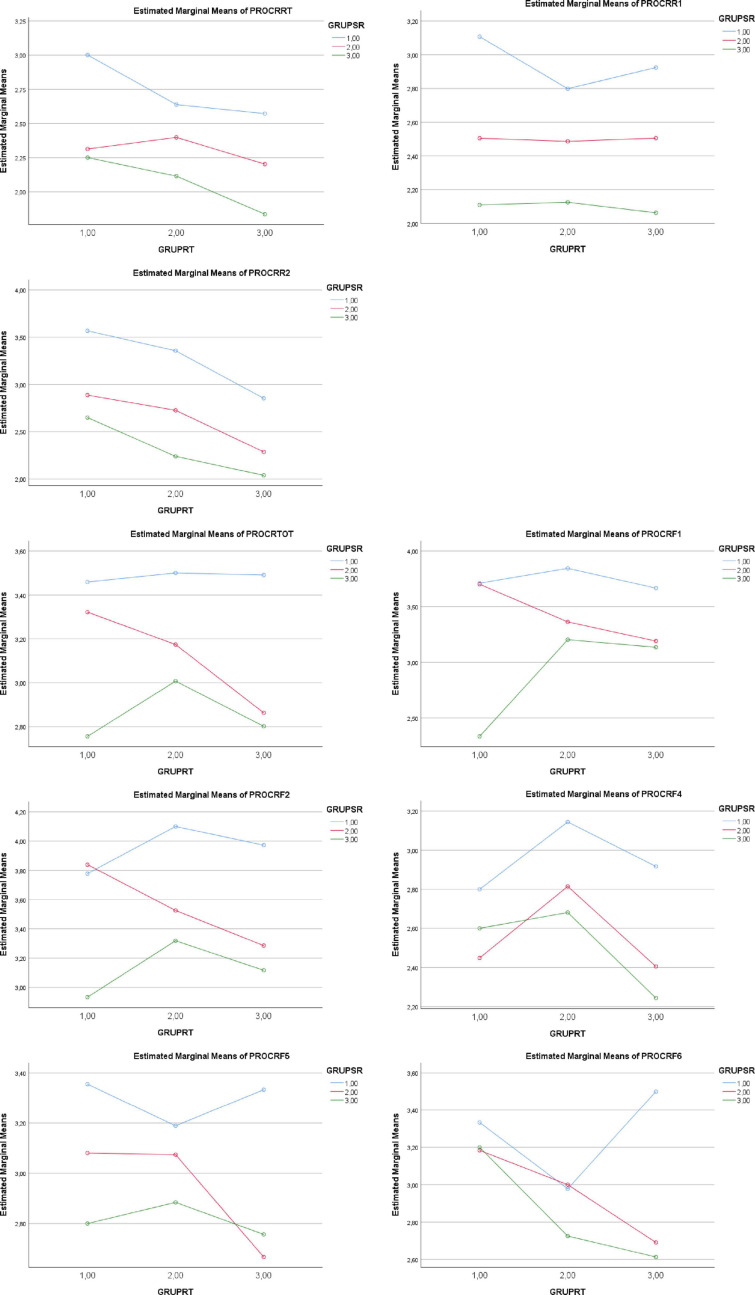
Graphic representation of the effect of *Self-Regulation* level (GRUPSR) and *Regulatory Teaching* level (GRUPRT) on *Reasons* and *Activities of Procrastination.* PROCRF1, term papers; PROCRATF2, study for exams; PROCRATF3, assigned reading; PROCRATF4, admin. tasks; PROCRASTF5, attendance; PROCRATF6, activities in general. Reasons for procrastination: PROCRATOT, TOTAL; PROCRAR1, AROUSAL SEEKING; PROCRR2, LOW CONTROL; PROCRATR3, PERFECTIONISM.

A statistically significant main effect of SR levels (1 = low; 2 = medium; 3 = high) was noted on total *Procrastination Activities* (1 > 2 > 3, *p* < 0.001). Complementarily, a statistically significant main effect of RT was noted on total *Procrastination Activities* (1,2 > 3, *p* < 0.001). Also, a statistically significant main effect of SR levels (1 = low; 2 = medium; 3 = high) was noted on the factors of *Procrastination Activities.* The main partial effects of SR levels (1 = low; 2 = medium; 3 = high) appeared in the procrastination activities of *writing a term paper*, *studying for an exam*, and *keeping up with weekly reading* (1 > 2 > 3, *p* < 0.001), while the RT levels (1 = low; 2 = medium; 3 = high) variable did not carry sufficient statistical strength to determine differences in any specific procrastination activity. See Table 2 and Figure 2.

### Combination Effects in Academic Behavioral Confidence and Procrastination

#### Preliminary Analysis

The MANOVA that was carried out showed statistically significant differences, in all levels of the SR and RT variables, among the five groups. SR and RT are adequately configured as established in Table 3.

#### Academic Behavioral Confidence

A statistically significant main effect of the *five combination of SR* levels *and RT* levels (see regulatory rank in Table 1) was noted on total *Academic Behavioral Confidence* (5,4 > 3,2 > 1; *p* < 0.001). Complementarily, a significant main effect of the *five combinations of SR* levels *and RT* levels was noted on the factors of *Academic Behavioral Confidence* (with variations of 5,4 > 3,2 > 1; *p* < 0.001). See Table 3 and Figure 1.

#### Reasons for and Activities of Procrastination

A statistically significant main effect of the *five combinations of SR* levels *and RT* levels was noted on total *Reasons for Procrastination* (1 > 2,3 > 4,5, *p* < 0.001). Regarding the factors of *Reasons for Procrastination*, a statistically significant main effect of the *five combination of SR and RT* was noted in all (1 > 2,3 > 4,5, *p* < 0.001).

**FIGURE 3:**
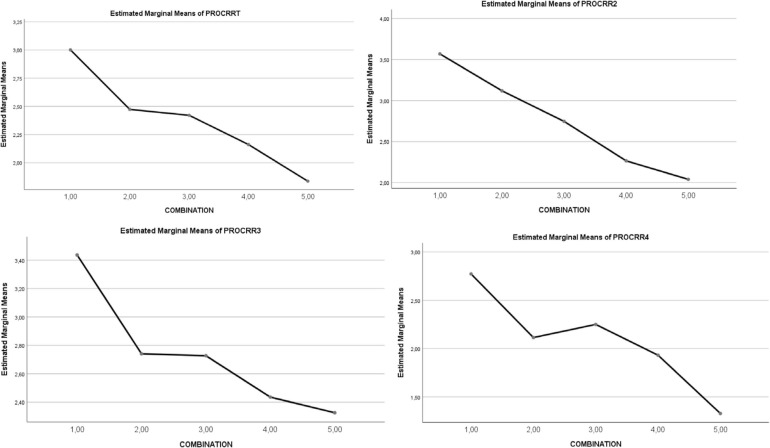
Graphical representation of the effect of combination types (1–5) on reasons to procrastinate. Reasons to procrastinate: RF1. AROUSAL SEEKING; RF2. LOW CONTROL; RF3. PERFECTIONISM; RF4. TEST ANXIETY; RF5. LOW CONFIDENCE.

**FIGURE 4:**
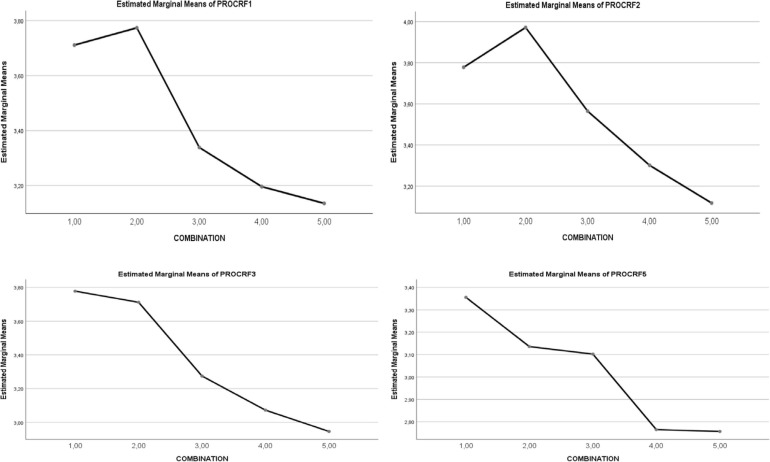
Graphical representation of the effect of combination types (1–5) on procrastination activities. Procrastination activities: F1. TERM PAPERS; F2. STUDY FOR EXAMS; F3. WEEKLY READING; F4. ADMINIST. TASKS; F5. ATTENDANCE; F6. ACTIV. IN GENERAL.

For total *Activities of Procrastination*, a statistically significant main effect of the *five combinations of SR and RT* levels was observed (1,2 > 3 > 4,5, *p* < 0.001). For all factors of *Activities of Procrastination*, a statistically significant main effect of the *five combinations of SR and RT* levels was noted, with particular statistical strength in *Writing term papers, Studying for exams* and *Keeping up with weekly reading* (1,2 > 3 > 4,5, *p* < 0.001). See Table 3 and Figures 3, 4.

### Structural Prediction Model

Pathway analysis (SEM) revealed an acceptable model of the relationships between variables. The relationship parameters of the two models are presented below. Both models were tested. In *model 1* the relationships Combination-> Academic confidence -> Activities of procrastination were tested, while in *model 2* the relationships Combination-> Academic confidence -> Reasons to procrastinate-> Activities of Procrastination The second model produced more consistent results and was taken as definitive. See Table 4.

**TABLE 4:** Models of structural linear results of the variables.

Chi^2^	*p*<	FG	CMFIN/FG	FI	RFI	IFI	TLI	CFI	HOELT	RMSEA
Model 1. 2229.258	0.001	242	9.211	719	0.835	0.843	0.860	0.810	0.189	0.103
Model 2.1097.968	0.001	135	8.12	0.908	0.913	0.907	0.926	0.906	0.206	0.085

#### Standardized Direct Effects

Of particular interest was the differential weight of SR (*B* = 0.62) and RT (*B* = 0.33) on the latent variable COMBINATION. The Model reflected that the combination of regulation factors (COMB) was a significant, positive predictor of academic behavioral confidence (CONFIDENCE) (*B* = 0.93). CONFIDENCE was also a significant, negative predictor of procrastination reasons (RAZPROCRAST) (*B* = −0.46) and procrastination activities (FACTPROCRAST) (*B* = −0.25). Finally, reasons for procrastinating appeared as a significant, positive predictor of procrastination activities (*B* = 0.32). See Table 5.

**TABLE 5:** Standardized direct effects (default model).

	COMBINATION	ACAD. BEH. CONFIDENCE	PROCRASTINATION REASONS	PROCRASTINATION ACTIVITIES
SELF-REGULATION	0.618			
REGULATORY TEACHING	0.331			
GRADES		0.813		
COMBINATION				
ACAD. BEH. CONFIDENCE	0.938			
REAS. PROCRASTINATION				
PROCRASTINATION ACT.			0.320	
VERBALIZATION		0.814		
ATTENDANCE		0.579		
STUDY		0.478		
R1. AROUSAL SEEKING			0.735	
R2. LOW CONTROL			0.624	
R3. PERFECTIONISM			0.809	
R4. TEST ANXIETY			0.623	
R5. LOW CONFIDENCE			0.808	
F1. TERM PAPERS				0.673
F2. STUDY FOR EXAMS				0.772
F3. ASSIGNED READING				0.891
F4. ADMINIST. TASKS				0.463
F5. ATTENDANCE				0.452
F6. ACTIVITIES IN GENERAL				0.597

#### Standardized Indirect Effects

The combination of SR and RT (COMBINATION) had statistically significant effects on the totals for procrastination reasons and procrastination activities and on their factors. Academic behavioral confidence (CONFIDENCE) also had an indirect negative, predictive effect on each of the factors and total of procrastination activities. See Table 6.

**TABLE 6:** Standardized indirect effects (default model).

	COMBINATION	ACADEMIC BEH. CONFID.	REASONS PROCRASTINATION	BEHAV. PROCRASTINATION
COMBINATION				
ACAD. BEH. CONFIDENCE				
REAS. PROCRASTINATION	−0.437	−0.147		
PROCRASTINATION BEH.	−0.377			
SELF-REGULATION				
REGULATORY TEACHING				
GRADES		0.771		
VERBALIZATION		:		
ATTENDANCE		.		
STUDY		.		
R1. AROUSAL SEEKING		−0.321	−0.338	
R2. LOW CONTROL	−0.273	−0.288		
R3. PERFECTIONISM	−0.353	−0.373		
R4. TEST ANXIETY		−0.272	−0.287	
R5. LOW CONFIDENCE	−0.169	−0.179		
F1. TERM PAPERS	−0.254	−0.268	0.215	
F2. STUDY FOR EXAMS	−0.293	−0.310	0.249	
F3. WEEKLY READING	−0.336	−0.354	0.285	
F4. ADMINIST. TASKS	−0.175	−0.184	0.148	
F5. ATTENDANCE	−0.170	−0.180	0.144	
F6. ACTIV. IN GENERAL	−0.225	−0.237	0.191	

**FIGURE 5:**
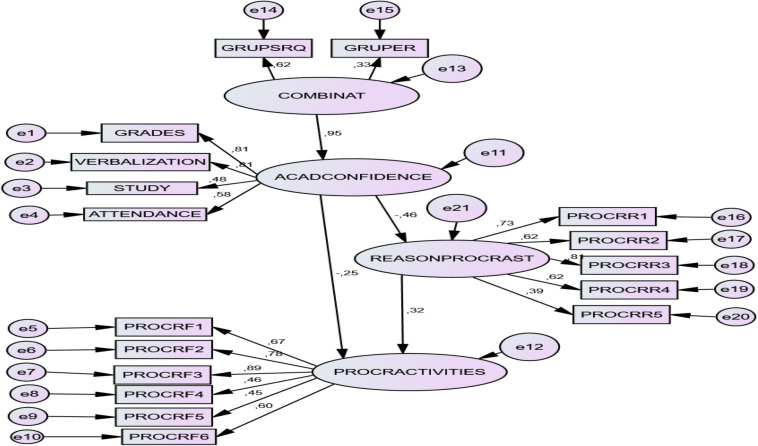
SEM of relations between academic behavioral confidence, reasons for procrastination and procrastination activities. COMBINAT, *SR and RT GROUPS*: ACADCONFIDENCE, *Academic Behavioral Confidence*; REASONPROCRAT, *Reasons to procrastinate*: RR1. AROUSAL SEEKING; RR2. LOW CONTROL; RR3. PERFECTIONISM; RR4. TEST ANXIETY; RR5. LOW CONFIDENCE. PROCRACTIVITIES, *Procrastination activities* (Factors): F1. TERM PAPERS; F2. STUDY FOR EXAMS; F3. WEEKLY READING; F4. ADMINIST. TASKS; F5. ATTENDANCE; F6. ACTIV. IN GENERAL.

A graphic representation of the final structural model is seen in Figure 5.

## Discussion and Conclusion

### Importance of the Level of Regulation Promoted Both Internally and Externally

Self- vs. Externally-Regulated Learning Theory (de la Fuente, 2017) had predicted that university students’ academic confidence and procrastination could be determined, jointly, by the students’ degree of *self-regulation* and by the level of contextual, *external regulation* from the teaching process. Furthermore, this type of interaction could be understood as the combination of the *low-medium-high level* of the two factors, and is supported by prior evidence in this direction, in reference to achievement emotions (de la Fuente et al., 2015b), to coping strategies used (de la Fuente et al., 2019) and to factors and symptoms of stress (de la Fuente et al., 2020c). In this study, in line with the hypotheses posed, the results contribute evidence that a *graded increase in level of regulation* (internal and external) gave rise to an increase in academic behavioral confidence, and a proportionate decrease in reasons for and activities of procrastination. By contrast, a graded decrease in level of regulation (internal and external) would lead to a decrease in academic behavioral confidence, and a proportionate increase in reasons for and activities of procrastination (Putwain et al., 2015; Putwain, 2018; Putwain and Pescod, 2018). We may consider that Hypothesis 1 was validated in almost every case. Both individually and in combination, levels of self-regulation (SR) and of regulatory teaching (RT) have produced an increase in academic behavioral confidence, as well as a decrease in procrastination reasons and activities. These results further our conceptualization of *academic behavioral confidence*, by showing that it depends not only on the university student’s level of regulation (de la Fuente et al., 2015b), but is also influenced by the level of regulation established in the teaching process. Specifically, the five-combination model (de la Fuente et al., 2019) is the most predictive model of variability in academic behavioral confidence (Sanders and Sander, 2003; Rusk et al., 2011; Saklofske et al., 2012).

*Hypothesis* 2 was also confirmed, establishing that regulation in students and regulation in teaching were both positive, significant predictors of academic behavioral confidence. Academic behavioral confidence, in turn, negatively predicted reasons for and activities of procrastination. Our linear predictive model revealed the same relationship in a structural format. It has been clearly shown that the combination of SR and RT predicts academic behavioral confidence, and that the latter directly and indirectly affects reasons for procrastinating and procrastination activites. Certain prior research studies have reported similar results, showing the predictive value of confidence with respect to procrastination in Secondary Education (Saputra et al., 2020). Klassen et al. (2008) showed that those who present more confidence in their academic skills (high levels of self-efficacy) procrastinate less. Given the results of the present study, there is evidence that academic behavioral confidence is determined not only by the student’s personal factors; and that academic behavioral confidence affects not only the intensity but also the types of procrastination (Brando-Garrido et al., 2020).

### Conclusion, Limitations and Future Research

Once again, consistent with the evidence reported in prior studies (de la Fuente et al., 2015b, 2017, 2019, 2020c, d), it has been confirmed that both the level of SR (in greater measure) and the level of RT produce effects on academic behavioral confidence, and on procrastination reasons and activities, and that the former is predictive of the latter. In a complementary way, it is possible to consider academic confidence as a protective factor against procrastination during university learning, since it minimizes the reasons and behaviors of procrastination (Batool, 2020).

One limitation of this study is the exclusive use of questionnaires for collecting data; obtaining another type of evidence from other data sources would make it possible to triangulate the information (Aguilar and Barroso, 2015), as well as corroborate and/or examine in more depth the findings presented here. A second limitation is the sample composition, which is predominantly female. For this reason, the sampling of participants may affect generalizability of the findings.

Future studies could address questions like the connection to previously reported variables with similar effects (health, flourishing, academic outcomes, etc.) in a model that integrates the cumulative evidence. In addition, further study could be made of the critical components of student self-regulation and of regulatory teaching, components that account for the important differences between the groups compared in this study. A clear understanding of these practices, habits and competencies would make it possible to develop guidance programs or classroom interventions that offer specific training in personal self-regulation and teaching regulation, and would promote application of these principles in educational contexts of university (Martín et al., 2003; Linnenbrink-Garcia and Pekrun, 2011; Lüftenegger et al., 2016; Shaw et al., 2017; Tada, 2017; Lekwa et al., 2018; Loderer et al., 2018; Mainhard et al., 2018; Shannon et al., 2019; McGee, 2020).

Interventions that seek to increase self-regulation or to decrease procrastination describe three types of action strategies: therapeutic treatment, therapeutic prevention and teacher/counselor intervention (Zacks and Hen, 2018). Along these lines, it is possible to develop non-therapeutic strategies in the academic context, for example, teacher- or counselor-led interventions to increase academic behavioral confidence, or interventions to improve the teacher’s external regulation skills. This type of strategy makes it possible to reach a larger student population, using a preventive approach (Freire et al., 2016, 2018; Frenzel et al., 2018).

### Implications for the Practice of Educational Psychology at University

These results once again confirm the importance of prior student variables (SR) in students’ academic behavioral confidence, and in their reasons for procrastination and procrastination activities. Hence the importance of understanding individual characteristics (Park and Adler, 2003; Moffa et al., 2016; Murayama et al., 2017; Pidgeon and Pittner, 2017) for preventing academic failure, and for carrying out counseling and educational guidance processes with university students (Parpala and Lindblom-Ylänne, 2012; Bhullar et al., 2014; Eckerlein et al., 2020).

One may also infer the need to intervene with teaching processes, offering training and guidance to help teachers design and develop more regulatory teaching processes, and reduce teaching processes that are non-regulatory or dysregulatory (Asikainen et al., 2014). Some meta-analytical studies (Schneider and Preckel, 2017) have indicated the importance of teacher-student interactions in academic achievement. After analyzing the effect of 105 variables on academic achievement, they found that the variable of teacher “availability and help” occupied the eleventh position, and “being friendly and respectful” with students occupied position 30. However, the present study shows that specific regulatory practices of teachers would have a positive impact on academic behavioral confidence, on reducing procrastination and on increasing students’ academic achievement, and can guide educational practice (Vermunt, 1989; Willcoxson et al., 2011; Villasana et al., 2016; Utriainen et al., 2018).

## Data Availability Statement

The raw data supporting the conclusions of this article will be made available by the authors, without undue reservation.

## Ethics Statement

The studies involving human participants were reviewed and approved by http://www.estres.investigacion-psicopedagogica.org/lib/pdf/CERTIFICADO_COMITE_DE_ETICA_UNAV.pdf. The patients/participants provided their written informed consent to participate in this study.

## Author Contributions

JF and AG-U contributed to conceptualization, design and analysis of data and contributed to first writing. PS wrote the final and revised the article. MV-M, SF, and MG contributed to data collection. All authors contributed to the article and approved the submitted version.

## Conflict of Interest

The authors declare that the research was conducted in the absence of any commercial or financial relationships that could be construed as a potential conflict of interest.

## Footnotes

1 www.inetas.net
